# Acoustic Voice and Speech Biomarkers of Treatment Status during Hospitalization for Acute Decompensated Heart Failure

**DOI:** 10.3390/app13031827

**Published:** 2023-01-31

**Authors:** Olivia M. Murton, G. William Dec, Robert E. Hillman, Maulik D. Majmudar, Johannes Steiner, John V. Guttag, Daryush D. Mehta

**Affiliations:** 1 Center for Laryngeal Surgery and Voice Rehabilitation, Massachusetts General Hospital, Boston, MA 02114, USA; 2 Speech and Hearing Bioscience and Technology, Division of Medical Sciences, Harvard Medical School, Boston, MA 02115, USA; 3 MGH Institute of Health Professions, Boston, MA 02129, USA; 4 Institute for Heart, Vascular, and Stroke Care, Massachusetts General Hospital, Boston, MA 02114, USA; 5 Department of Surgery, Harvard Medical School, Boston, MA 02115, USA; 6 Biofourmis, Inc., Boston, MA 02110, USA; 7 Division of Cardiovascular Medicine, Oregon Health & Science University, Portland, OR 97239, USA; 8 Computer Science & Artificial Intelligence Laboratory, Massachusetts Institute of Technology, Cambridge, MA 02139, USA

**Keywords:** congestive heart failure, voice and speech biomarkers, daily monitoring

## Abstract

This study investigates acoustic voice and speech features as biomarkers for acute decompensated heart failure (ADHF), a serious escalation of heart failure symptoms including breathlessness and fatigue. ADHF-related systemic fluid accumulation in the lungs and laryngeal tissues is hypothesized to affect phonation and respiration for speech. A set of daily spoken recordings from 52 patients undergoing inpatient ADHF treatment was analyzed to identify voice and speech biomarkers for ADHF and to examine the trajectory of biomarkers during treatment. Results indicated that speakers produce more stable phonation, a more creaky voice, faster speech rates, and longer phrases after ADHF treatment compared to their pre-treatment voices. This project builds on work to develop a method of monitoring ADHF using speech biomarkers and presents a more detailed understanding of relevant voice and speech features.

## Featured Application

A key potential application of the current work is prospective at-home monitoring of patients at risk for acute decompensated heart failure using non-invasive acoustic voice and speech biomarkers, which can help avoid rehospitalization and reduce morbidity and mortality.

## 1. Introduction

Heart failure (HF) is the primary diagnosis for over 1.1 million hospitalizations each year in the United States [1], and is a leading cause of hospitalization in patients over 65 years of age (prevalence estimated to be 9–12%) [2]. Worldwide, it was reported for the year 2017 that nearly 65 million people were estimated to be living with heart failure [3,4]. In individuals with HF (either reduced or preserved ejection fraction), increased filling pressures and/or impaired cardiac output can spark a cascade of compensatory mechanisms to maintain adequate blood supply to the body. In the long term, these alterations to homeostasis can lead to further deterioration of heart function that cannot be compensated for [5]. Individuals with HF can remain stable and out of the hospital for long periods of time but can also deteriorate into acute decompensated heart failure (ADHF) [6]. In ADHF, excess fluid or congestion can occur in the lungs or systemically throughout the body, especially in the lower limbs [7]. Each hospitalization for ADHF increases the risk of subsequent hospitalization and overall mortality [6]. After discharge from a hospitalization for ADHF, 24% of patients are readmitted within 30 days. As many as 75% of these 30-day readmissions may be preventable, with increased follow-up and monitoring in the immediate post-discharge phase appearing to be particularly effective in reducing readmissions [8]. Preventing ADHF, and associated hospitalization, is therefore a major goal for patients with HF.

Patients with HF are often advised to track unexpected increases in their body weight at home to detect impending decompensation, since increased fluid volume due to congestion can increase weight. However, for monitoring to be useful in averting hospitalization, decompensation must be detected early enough to allow time for clinical intervention. Changes in weight, clinical symptoms, and/or cardiac resynchronization therapy parameters (if available) are not always reliable enough to allow this early detection [8]. Intravenous pulmonary artery pressure monitoring has revealed that congestion starts to increase weeks before clinical symptoms appear or worsen to the point of requiring hospitalization, but is invasive and expensive [8]. There is currently an unmet need for a reliable, non-invasive, early warning monitoring system for an ADHF episode. The finding that there are pre-symptomatic increases in congestion suggests an immediate use for monitoring techniques that detect sub-clinical changes in physiological status.

### 1.1. Linking Voice Physiology to ADHF

Voice is produced when air flows from the lungs past the vocal folds, which are held in an approximately closed position. The pressure difference above and below the vocal folds forces bursts of air through them, creating a periodic signal. Biomechanical properties of the vocal folds affect their readiness to vibrate in response to air pressure from the lungs [9]. The phonation threshold pressure (PTP) is defined as “the minimum lung pressure required to initiate phonation” [10]. The relevant biomechanical vocal fold properties include tissue elasticity, thickness, and viscosity [11], which is closely linked to hydration level in the vocal folds [12].

PTP and hydration level have both been linked to phonatory effort. In [13], healthy adults were given the diuretic Lasix (furosemide), which decreases systemic hydration. For participants who received the diuretic, PTP increased by 23% within 5 to 12 h of treatment. In contrast, control speakers not given the diuretic showed no increase in PTP during the same period. That study is particularly relevant since Lasix is also widely used to treat decompensated HF [6]. Additionally, several acoustic measures of vocal perturbation (jitter, shimmer, and signal-to-noise ratio) have been found to improve in response to hydration treatments for speakers with laryngeal nodules or polyps [14]. These speakers also reported reduced phonatory effort after hydration treatments. Other studies have found that dehydration can reduce vocal quality, and conversely, that increasing hydration can improve jitter, shimmer, and maximum phonation times [15].

**Figure 1.**
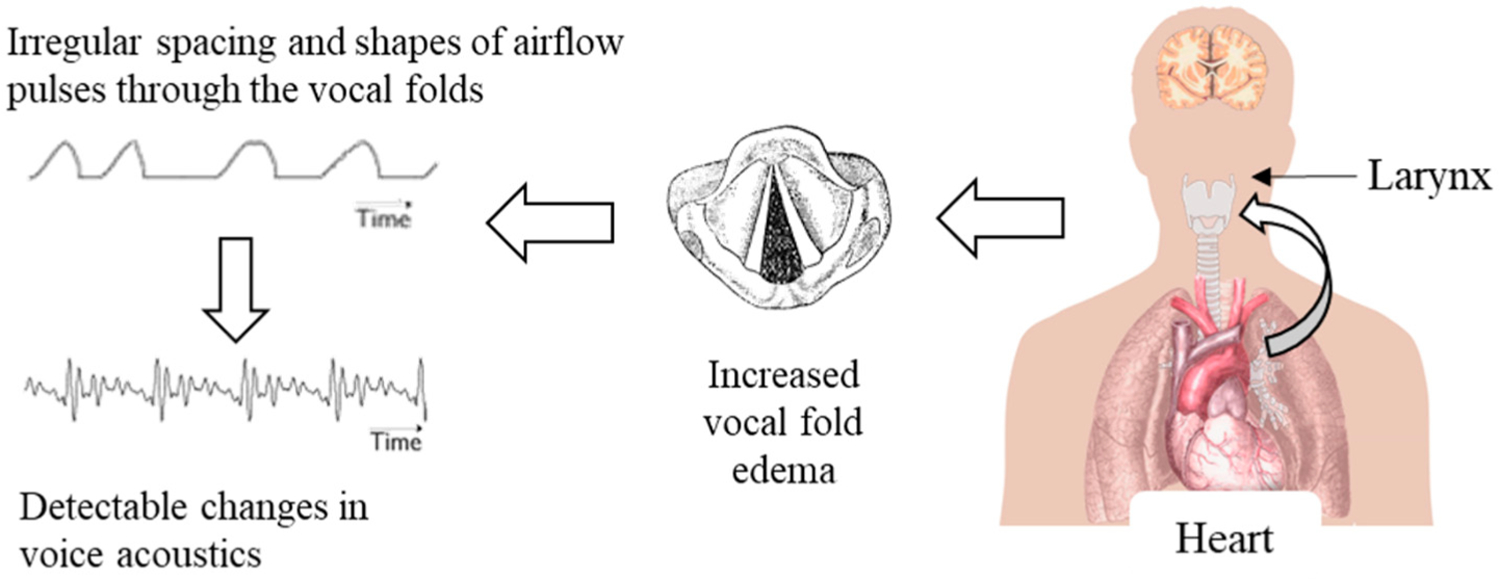
Hypothesized effects of HF-related congestion on laryngeal edema, voice airflow, and properties of the acoustic voice signal, whose features have the potential to be detectable earlier than systemic increases in body weight.

The findings linking hydration to PTP and vocal effort indicate a direct relationship among systemic fluid levels, vocal fold physiology, and vocal function. These results suggest that HF-related congestion and edema, if present in the vocal folds, could also affect the resulting voice signal in detectable ways. The amount of laryngeal edema required to measurably change the voice is expected to be small, especially in contrast to the large amount of systemic edema needed to produce an easily detectable increase in body weight. Therefore, voice monitoring may allow patients and clinicians to detect and track HF-related congestion at an earlier stage than weight monitoring does. This sequence of events is summarized in Figure 1.

### 1.2. Voice and Speech as Biomarkers of Health

Characterizing voice and speech characteristics provides an accessible, non-invasive way to monitor physiological changes throughout the body, both for voice disorders and as a biomarker of general health [16,17]. Especially in recent years, voice and speech biomarkers have been developed to monitor several laryngeal, neurological, and psychological disorders. These include vocal hyperfunction [18], depression [19], Parkinson’s disease [20,21], and amyotrophic lateral sclerosis [22]. Voice has also been increasingly used as a biomarker for cardiovascular and respiratory disorders, including coronary artery disease [23], pulmonary hypertension [24], and COVID-19 [25].

Maor et al. [26] developed a vocal biomarker that predicted hospitalization and mortality in patients with HF. The biomarker was developed from a cohort of over 8000 speakers with chronic conditions other than HF, including cancer, lung disease, and diabetes. Acoustic voice features relating to the cepstrum, pitch, loudness, and perturbation were extracted from 20 s voice samples. A large set of abstract, high-level features was extracted from the time series of these acoustic features, and the resulting data set was used to train a linear biomarker model to predict all-cause mortality. When tested on a separate cohort of 2200 speakers with HF, the biomarker was associated with the risk of both hospitalization and mortality. Each increase of one standard deviation in the biomarker represented an increase in risk of 48% for death and 25% for hospitalization during the 20-month follow-up period. This study strongly suggests that the pathophysiology of HF causes clinically relevant changes in the voice, although the specific mechanisms that governed the relationship between voice and HF status were not investigated and are still unknown. Maor et al. did not identify specific voice features that were well-correlated with the risk of hospitalization or mortality. However, in that and related work [24], changes in laryngeal perfusion, impaired vagus nerve functioning, physical compression of the left recurrent laryngeal nerve, and increased emotional distress are implicated as possible causes of voice changes due to cardiopulmonary disease.

In a recent study, five speech measures were analyzed from 40 patients with ADHF at admission and discharge [27]. The specific acoustic measures were not reported but showed good accuracy in distinguishing voice samples from admission versus discharge. In a particularly novel contribution, Amir et al. [27] included an auditory-perceptual evaluation in which 20 listeners classified seven voice samples from each of three patients with ADHF as either “wet” (having increased fluid levels on admission) or “dry” (having fluid levels return to baseline at discharge). Listeners were able to correctly classify these samples in 67% of cases, which was statistically above the 50% chance rate. These results, albeit very preliminary due to a small sample size, indicate that voice changes in heart failure may be significant enough to be detected perceptually as well as acoustically, which is the focus of the current line of research.

### 1.3. Current Work

This investigation builds upon the pilot study presented in [28] with several advancements. The initial data set of 10 pilot participants has been expanded to include recordings from 52 patients undergoing inpatient treatment for ADHF. As in [28], patients’ voices were compared at admission (pre-treatment) and discharge (post-treatment) based on a set of features relating to voice quality and respiratory support for voice and speech production. Due to the expanded sample size, in this study, we used those features to train logistic classifiers that distinguished voice samples from admission and discharge. The expanded sample size and more-sophisticated statistical analysis represent a significant increase in scope over our previous pilot work. In contrast with previous work on vocal biomarkers for HF [26], the trained classifiers were also used to identify which voice and speech features had the greatest predictive power to differentiate pre- and post-treatment recordings. In addition, we computed the probability of discharge for each daily recording and examined the trajectories of these day-to-day changes. We hypothesized that voices from speakers at discharge (after ADHF treatment) would show increased pitch, increased vocal stability, and improved respiratory support compared to their pre-treatment baselines.

## 2. Materials and Methods

### 2.1. Participants

Using convenience sampling, we enrolled fifty-two participants (20 female and 32 male) who were hospitalized for multiple days due to a diagnosis of ADHF at Massachusetts General Hospital in Boston, MA (40 participants) and at the University of Vermont Medical Center in Burlington, VT (12 participants). It is noted that the literature indicates that the prevalence of heart failure is similar across sexes [29,30]. Thus, future studies could balance enrollment and take into account sex in the analysis. Procedures followed were in accordance with the ethical standards of the institutional review board at Mass General Brigham (protocol #: 2015P000986, most recent approval date: 15 April 2022). Inclusion criteria called for patients to have pre-existing diagnoses of chronic HF and current diagnoses of ADHF requiring at least 48 h of diuresis. Patients were also required to be above their “target weight,” which refers to their typical body weight without HF-related extra fluid volume. Exclusion criteria included respiratory infection, pulmonary disease, kidney disease, and history of a voice disorder. The participants had a median age of 72 years (range of 34–96 years) and median baseline ejection fraction of 44% (range of 12–78%).

At both enrollment sites, patients were enrolled in the study as soon after their admission as possible, at most 24 h after admission. Each day of hospitalization, patients were weighed and asked to perform a standard speech protocol. Since high levels of NT-proBNP are associated with HF [31], blood levels of N-terminal pro b-type natriuretic peptide (NT-proBNP) were tested at the beginning and end of each patient’s hospitalization. Patients evaluated their dyspnea symptoms and global symptoms using visual analog scales with endpoint anchors at 0 (worst) and 100 (best). The mean length of hospitalization was 7.5 (range of 2 to 32) days, median weight change (last minus first measurement) was −5.5 (range of −24.9 to 4.8) kg, and median change in NT-proBNP level was −567 (range of −9200 to 57,400) pg/mL. The median changes in participants’ ratings of dyspnea and global symptoms (last minus first ratings) were, respectively, 11.5 (range of −43 to 67) percentage points and 16 (range of −20 to 68) percentage points.

### 2.2. Data Collection

A speech protocol was performed on the day of enrollment and then approximately one time per day of hospitalization until discharge, up to a maximum of ten recordings. If a patient was expected to be hospitalized longer than ten days, recordings were less frequent so that their ten recordings could span the entire course of their treatment through discharge. When possible, each patient’s last recording was done on the day of, or day before, their discharge.

The standard voice recording protocol included a maximum phonation time (MPT) test, three/a/vowels each 3–5 s in duration, six standard voice assessment sentences from the Consensus Auditory-Perceptual Evaluation of Voice form [32], the Rainbow Passage [33], a second reading passage, and 30 s of spontaneous speech. The second reading passage was chosen from a set of 10 such that each participant read a new passage each day and was used to control for participants gaining familiarity with the daily Rainbow Passage. All passages were edited to a Flesch–Kincaid reading level of approximately grade 6. The MPT and second reading passage tasks were added midway through data collection, therefore only a subset of participants performed those tasks. Collectively, the 52 speakers performed 3004 vocal tasks over 255 recording sessions.

Both a microphone (MIC; H1 Handy Recorder, Zoom Corporation, Tokyo, Japan) and neck-mounted accelerometer (ACC; model BU-27135, Knowles Corp., Itasca, IL, USA) were used to record participants’ speech [34]. The accelerometer preserves speaker privacy and is more robust to background noise than the acoustic microphone, making it particularly useful for ambulatory recordings. Each session was recorded with the microphone and accelerometer, generating one MIC and one ACC recording per participant per day. MIC recordings were sampled at 44,100 Hz, and ACC recordings were sampled at 11,025 Hz. All recordings were resampled to 25,000 Hz to allow for time alignment of both sensor signals. To remove low-frequency noise artifacts, MIC recordings were high-pass filtered at 70 Hz (Hann band-pass filter, 100 Hz smoothing) using Praat [35].

### 2.3. Feature Extraction

Frame-by-frame fundamental frequency (F0), creak, and cepstral peak prominence (CPP) contours were computed for each single-task audio file. Praat was used to generate a voice report to determine additional measures of vocal stability, and speech phrase durations were used as measures of respiratory capacity [35]. Finally, MPT was calculated as the duration between the first and last voiced frames for each MPT task recording. Most features were extracted as described in [28], with the following exceptions.

Measures of speech phrase duration were computed for the continuous speech tasks. Within an utterance, pause durations longer than 500 ms were assumed to contain an inhalation [18]. A speech phrase was defined as the speech between two successive inhalations. In other words, voiced regions separated from each other by less than 500 ms were considered to be part of the same speech phrase. Measures of breath capacity that were computed following this method included the number of speech phrases, total duration of all the speech phrases, and mean, median, and standard deviation (SD) of the speech phrase durations in each utterance.

F0 was extracted with Praat’s cross-correlation pitch-tracking method, which uses a 40 ms Hanning window every 3.3 ms. To minimize distortion from tracking artifacts, only frames with F0 in the 5–95th percentile range for a task were used to compute mean F0, median F0, and F0 SD. For sustained vowel tasks, the F0 SD computation was further restricted to frames with F0 within 50 Hz of the median F0. Using this 100 Hz range allowed the F0 SD calculation to include normal pitch fluctuations while reducing the effect of tracking errors that undesirably doubled or halved the true F0. Other acoustic perturbation features derived from the Praat voice report (jitter, shimmer, harmonics-to-noise ratio, and the low–high spectral energy ratio) were computed from the center 500 ms of each vowel to avoid instability from voicing onsets and offsets.

For each task, the probability of creak, an acoustic feature related to irregularity and vocal fry perception, was computed every 10 ms using a method developed by [36] and [37]. Acoustic features relating to short-term power contours, intra-frame periodicity, inter-pulse similarity, and the presence of secondary or widely spaced glottal pulses were used as inputs to an artificial neural network that outputs frame-by-frame creak probabilities. Here, frames were considered voiced if they (1) had non-zero F0 based on the Praat pitch track, (2) occurred within a speech phrase, or (3) had a creak probability above 0.8. Adding high-probability creak frames additionally accounted for frames with highly irregular phonation—often on the margins of voiced regions—where Praat’s pitch tracking did not identify an underlying F0. For each utterance, the creak percent was given by (# creaky frames)/(# voiced frames) × 100. The “# creaky frames” quantity was defined as voiced frames with creak probability either above 0.02 [38], or, separately, above 0.3 [39].

Cepstral peak prominence (CPP) was used as a measure of degraded voice quality or dysphonia, with higher CPP indicating healthier voice quality [40]. CPP and CPP SD were calculated for both vowel and Rainbow Passage utterances following [41]. The CPP was required to fall between quefrencies of 3.3 and 16.7 ms, which are equivalent to F0 between 60 and 300 Hz. In that quefrency range, CPP was given by the difference between the magnitude of the highest peak and the power cepstrum’s noise floor (window length 40.96 ms, computed every 10.24 ms). CPP was reported only for frames that met one of the three voicing criteria used for the F0 measures: frames with positive F0, within a speech phrase, or with creak probability above 0.8. For each task, frames with CPP between the 5th and 95th percentile were used to compute mean, median, and SD of CPP.

### 2.4. Statistics and Machine Learning

Analyses were carried out separately for the microphone-based recordings and for the accelerometer-based recordings. When a participant performed the same task multiple times on a single day, the measures based on that task were averaged to produce a single value per measure per day. For each measure, Cohen’s *d* effect sizes for paired samples were computed as $\frac{{\bar{x}}_{2}-{\bar{x}}_{1}}{\sigma }$, where ${\bar{x}}_{1}$ and ${\bar{x}}_{2}$ were the means of the admission and discharge values for each measure, respectively, and *σ* was the standard deviation of the admissionto-discharge day differences.

To investigate whether voice and speech features could distinguish stable versus decompensated heart failure, we used machine learning to classify speech samples into “admission” and “discharge” classes. Patients’ first recordings were labeled as “admission”, and their last recordings were labeled as “discharge”. We used supervised learning to train a classifier based on a regularized logistic regression model, with the acoustic voice and speech features described above as inputs and the admission/discharge classes as labels to be predicted. This classification task was chosen as it allows for an initial understanding of how well voice and speech features may perform on a binary healthy/unhealthy classification task that could then be used to inform a more granular analysis of feature trajectories.

Each data point for the model input consisted of the admission or discharge label and all the features associated with a single day of recording for a single participant. There were thus 104 input points for each of the 52 participants (features at admission and discharge). Some features could not be computed for every recording, since occasionally participants did not complete a task or the recording quality was inadequate for analysis. Any data point with one or more features missing was excluded from the model input. The remaining input data was z-normalized so that each feature had a mean of 0 and standard deviation of 1. Since the MPT was added partway through data collection, not all participants completed the task. Therefore, the model creation process was run twice: once with all the participants but not the MPT feature (“MPT−” model) and once with the MPT feature but only the participants who completed it (“MPT+” model). After removing missing data, the MPT− model had 91 inputs from 51 participants, and the MPT+ model had 42 inputs from 24 participants.

MATLAB’s *fitclinear* function was used to train logistic classifiers using the leave-one-participant-out cross-validation method described below. In these classification models, *n* input features {*x*_1_, *x*_2_, … *x*_*n*_} are combined linearly using learned coefficients {*β*_0_, *β*_1_, *β*_2_, … *β*_*n*_}:

(1) $$f(x)=\beta_{0}+\beta_{1}x_{1}+\beta_{2}x_{2}+\ldots +\beta_{n}x_{n}$$

The learning process attempts to find *β* coefficients that minimize the loss function *L* for a prediction relative to the labels {*y*_1_, *y*_2_, ⋯ *y*_*n*_} that indicate whether a data point was produced at admission (+1) or discharge (−1):

(2) $$L(y,f(x))=\text{log}\left(1+e^{-yf(x)}\right)$$

where *y* is the known label (+1 or −1) and *f* (*x*) is the model output, which ranges from +1 to −1.

Intuitively, this loss function penalizes large mismatches between the label *y* and the model output *f* (*x*) for each point. It especially penalizes predictions that have opposite sign to the labels. When *y* and *f* (*x*) have the same sign for a given data point, the model’s classification is correct: the prediction correctly aligns with that point’s label. In that case, the product −*yf* (*x*) is negative, and *e*^−*yf*(*x*)^ < 1. If *y* and *f* (*x*) do not have the same sign (i.e., if the classification is incorrect), then −*yf* (*x*) > 0, and *e*^−*yf*(*x*)^ > 1.

These models were trained and tested using the following variant of leave-one-out cross-validation. For each cross-validation fold, the model was trained on data from all but one participant and then tested on the held-out participant’s data. The leave-one-participant-out approach maximizes the amount of training data available in each fold, but also prevents the model from training and testing on the same participant’s data.

Leave-one-participant-out cross-validation sees each participant in the test set exactly once, so a single prediction is made for each data point. The cross-validated model’s performance was evaluated by calculating the proportion of recordings correctly classified as at admission (first day) or discharge (last day), the area under the receiver operating characteristic curve, sensitivity, and specificity. Confusion matrices were also generated to visually inspect model performance. The logistic regression model was also used to identify the features that best discriminated between voices at admission and discharge. The odds ratios for each feature coefficient *β*_i_ was calculated as $e^{\beta_{i}}$. An odds ratio farther from 1 (either above or below) indicates greater predictive power for that variable.

This data set had many features relative to the number of data points, so there was a high risk of overfitting causing poor test accuracy. To reduce overfitting, the model fitting process used L1 (or lasso) regularization [42]. Without regularization, the learner finds feature coefficients that minimize classification loss. In L1 regularization, a term given by the sum of the absolute values of the coefficients is added to the loss function:

(3) $$\underset{\beta }{\text{min}}\frac{1}{n}\sum_{i}^{n}\text{Loss}\left(y_{i},f\left(x_{i}\right)\right)+\lambda \sum_{j}^{m}\left|\beta_{j}\right|$$

This regularization term penalizes large coefficient weights. L1 regularization performs a form of feature selection: as λ increases, more coefficient weights are set to zero. Adding this regularization term can improve test accuracy: with fewer features involved in predicting outputs, it is harder to overfit to the training data. If λ is too large, accuracy decreases: many features are discounted from the model and not enough remain to capture the data’s patterns. Eventually, for some large-enough value of λ, all weights are zero and the model simply predicts the most common class for all inputs. For each model, we used *fitclinear* hyperparameter optimization to sweep 30 logarithmically spaced λ values in the range [0.001, 1]. The resulting models were used to identify the λ that yielded the lowest cross-validated loss.

## 3. Results

**Figure 2.**
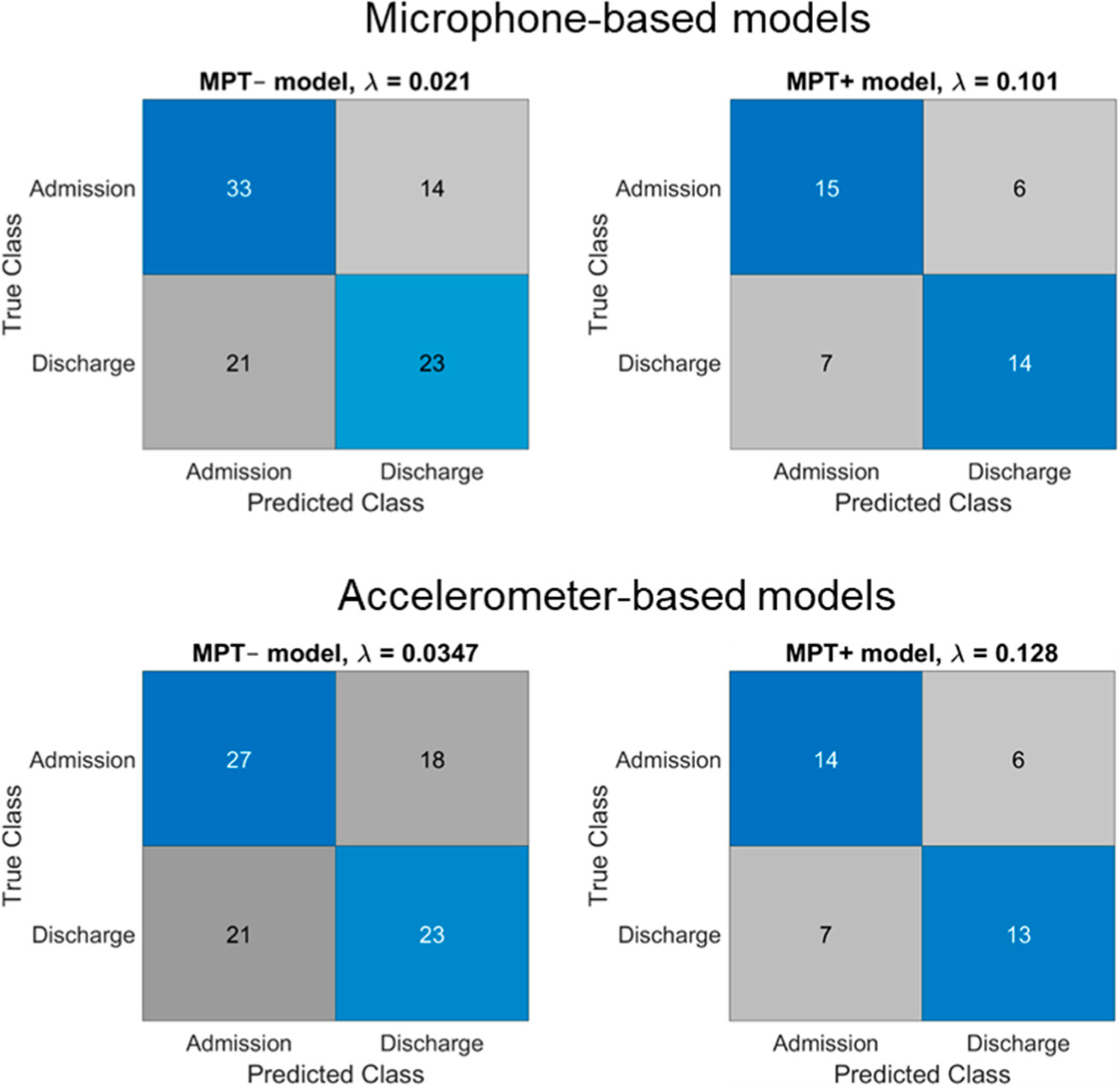
Confusion matrices and optimized lambda values (λ) for the microphone-based and accelerometer-based models without (MPT−) and with (MPT+) the maximum phonation task.

Effect sizes for total phrase duration, MPT, F0 mean, CPP mean, CPP median, and CPP SD are reported in Table 1. Effect sizes are reported for both MIC- and ACC-based data and are sorted in decreasing order of magnitude for MIC-based data. Figure 2 shows confusion matrices for the MPT− and MPT+ models. Quantities in blue-shaded (upper-left and lower-right) squares indicate the number of data points correctly classified as admission or discharge. Grey-shaded (upper-right and lower-left) squares indicate incorrectly classified points.

These results represent the average performance of the models created during cross-validation. However, it is also desirable to identify a single set of feature weights, since these weights can provide information about each feature’s predictive power. To identify a single set of feature weights, we trained new MPT− and MPT+ models without cross-validation, using all the training data. The resulting odds ratios (given by *e*^*β*^, where *β* is the feature weight) are listed in Tables 2 and 3. These normalized odds ratios for each feature indicate the change in the probability of discharge when values for that feature increase by one standard deviation. An odds ratio farther from 1 indicates that a feature makes a greater contribution to the classification decision.

The models’ performances were evaluated based on their accuracy, area under the receiver operating characteristic curve, true admission rate (sensitivity), true discharge rate (specificity), admission predictive value (positive predictive value), and discharge predictive value (negative predictive value). “Admission” and “discharge” are used in place of “positive” and “negative” in the names of these metrics to increase clarity. Performance metrics for the five models are summarized in Table 4.

**Figure 3.**
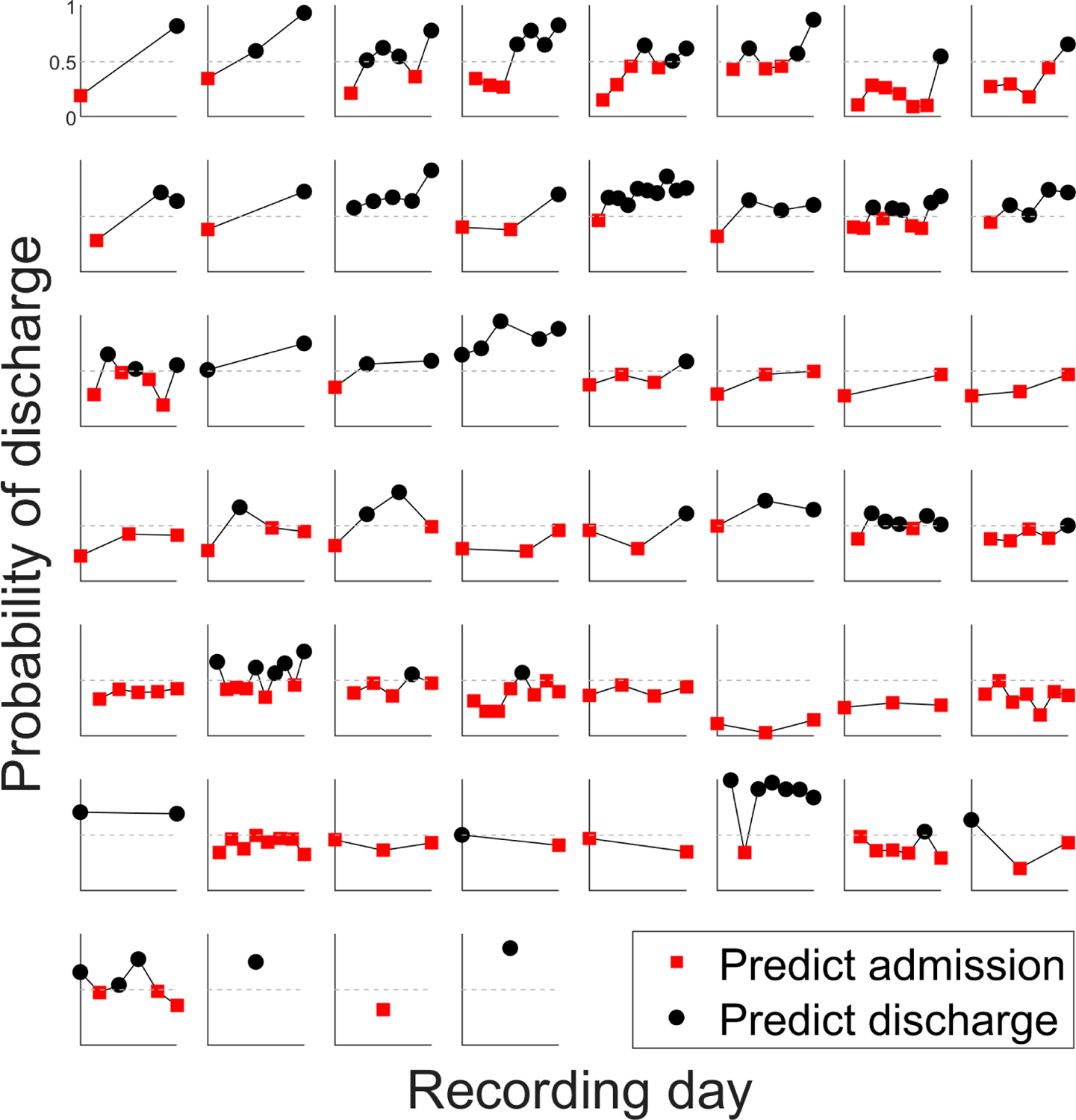
Day-to-day discharge probabilities for each patient based on the microphone-based MPT− model, with daily recording sessions on the horizontal axis of each subplot. Red squares indicate admission predictions (discharge probabilities below 0.5 threshold [dashed line]), and black dots indicate discharge predictions (discharge probabilities > 0.5). Participants are ordered (**left** to **right**, **top** to **bottom**) from the greatest to least first-to-last-day change in discharge probability.

The MPT− and MPT+ models, which were trained on all of the participants’ data, were also used to generate the probability of discharge for every daily recording from each individual rather than simply the recordings from admission and discharge days. This follow-up visualization generated a series of day-to-day discharge probabilities for each participant. These trajectories are shown in Figure 3. Note that three participants only yielded recordings from a single day due to either patient non-compliance or scheduling issues. Other participants yielded multiple days or recordings according to their treatment schedule. The trajectories from the 52 participants are ordered from the greatest to least first-to-last-day change in discharge probability. Within each plot, a participant’s recordings are ordered sequentially from admission to discharge. If the probability of discharge for a point was below 0.5, the point was classified as “admission” and plotted in red. Black points indicate recordings for which the discharge probability was above 0.5. In general, the probability of discharge increased over the course of the hospital stay. At the same time, most participants showed day-to-day fluctuations outside of that overall trajectory. Follow-up work is warranted to correlate changes in the discharge probability with clinical metrics obtained during each day.

## 4. Discussion

The findings presented above indicate that acoustic speech features have the potential to distinguish ADHF patients’ pre- and post-treatment speech samples with an accuracy up to 69%, sensitivity up to 71%, and specificity up to 67% (Table 4). The acoustic features that best predicted that a sample was after treatment were higher MPT, more stable phonation (decreased F0 SD in sustained vowels, increased CPP, and decreased CPP SD), increased creak percent, faster speech rate, and reduced pausing compared to voice and speech characteristics before treatment. These features are hypothesized to reflect physiological changes in the larynx and lungs due to fluid accumulation from ADHF. This interpretation is tempered by the presence of false positives and false negatives output by the applied machine learning models. However, these sorts of models can be used as a screening tool that can aid in quantifying the potential for a heart-related condition/episode in conjunction with other symptoms that are either self-reported by an individual or measured by clinical staff. Future work with augmented data sets could benefit from ensemble learning in which different models could be learned from data stratified by patient severity.

Importantly, these results are based on speakers who were recovering from ADHF and were receiving inpatient treatment. The long-term goal of this work is to monitor stable HF patients at home to predict and prevent ADHF episodes before they require hospitalization. Therefore, it is important to determine whether these voice changes (or similar ones) are also present in speakers who are developing ADHF. If they are, then patients and clinicians could use at-home voice monitoring to assess ADHF risk and guide outpatient treatment. These results are also based on a short set of speech tasks (i.e., reading and sustained vowel tasks), most of which did not involve spontaneous speech. Future work might reveal that longer or more natural speech samples provide better information about vocal function and ADHF risk. In that case, a neck-surface ACC sensor could be used for ambulatory voice monitoring to evaluate ADHF risk based on real-world voice use [43].

Due to the sample size, the statistical approach applied was one of supervised machine learning with leave-one-out cross-validation. This approach was selected as an initial window into the performance of the features and models investigated that reflect voice and speech properties of patients with acute decompensated heart failure (Table 4). Future work would benefit from a much larger sample size to allow for a separate held-out data set for testing model performance.

### 4.1. Promising Acoustic Voice and Speech Features

#### 4.1.1. Total Phrase Duration

For a continuous speech task, the “total phrase duration” feature was given by the sum of the durations of all the speech phrases in that utterance. In other words, this measure represents the total duration of the recording minus any pause time. The effect size (Cohen’s *d*) for this measure was −0.5 for both the Rainbow Passage and the second reading passage. This value indicates that total phrase duration tended to decrease from admission to discharge, with a moderate effect size [44]. The Rainbow Passage text did not vary from day to day, and the second reading passages were all approximately equal length and presented in random order. A decrease in the amount of time needed to read these texts, then, indicates that participants were able to read faster at discharge compared to admission.

In our pilot study [28], total phrase duration in the Rainbow Passage decreased from admission to discharge for most participants. In that study, participants read the same text each day. Therefore, we initially hypothesized that the decrease in total phrase duration may have been a practice effect: participants were able to read the text more quickly because they were more familiar with it. We later added the second passage-reading task to control for any familiarity effect. Since there were 10 additional reading texts, participants read an unfamiliar one each day. Total phrase duration also decreased for this second reading task, with an equally large effect size (−0.5) as for the Rainbow Passage task. This finding indicates that participants’ ability to read faster at discharge is not due only to familiarity with the reading text. In future work, it may also be useful to look at the speech rate in the spontaneous speech tasks, since that task would not depend on the speaker’s reading ability.

In a relevant finding, Ramig [45] found that, especially among older speakers, speaking and reading rates were correlated with overall level of health. Similarly, Linville et al. [46] examined relationships between various speech production measures in women aged 67 and older. They found that reduced reading rate correlated with increases in phonatory instability, including jitter and F0 SD. They suggest that “generalized loss of physiological control” could alter both reading rate and phonatory stability. In their case, the suggested change in physiological control was primarily related to age, but HF-related changes in physiological status might similarly affect vocal control. In particular, Linville et al. [46] found a “significant correlation” between reduced reading rate and increased vocal fold edema on a laryngoscopic exam. The causes of their speakers’ vocal fold edema were not specified, but this finding strongly suggests that HF-related vocal fold edema may have direct physiological effects on voice and speech measures, including speaking and reading rate.

#### 4.1.2. Maximum Phonation Time

The effect size for MPT was moderate, at 0.49 [44]. This is a promising finding: MPT is related to phonatory ability and breath support, both of which we hypothesized would be affected by HF-related edema. It is important to note that the mean MPT even at discharge, when speakers were at their healthiest, was only approximately 10 s. In contrast, Maslan et al. [47] measured MPT in healthy adults aged 65 years or older and found that MPT did not decrease with age alone in the absence of comorbidities; i.e., healthy older adults had similar MPTs as younger adults. In healthy speakers aged 60–90 years, they found a mean MPT of 22 s, which is substantially longer than the mean 10 s MPT for our speakers at discharge. It is important to keep in mind that the admission versus discharge classification reported on is not the same as identifying whether someone is healthy or has HF. This study is only aimed at differentiating degrees of severity within already diagnosed individuals.

**Figure 4.**
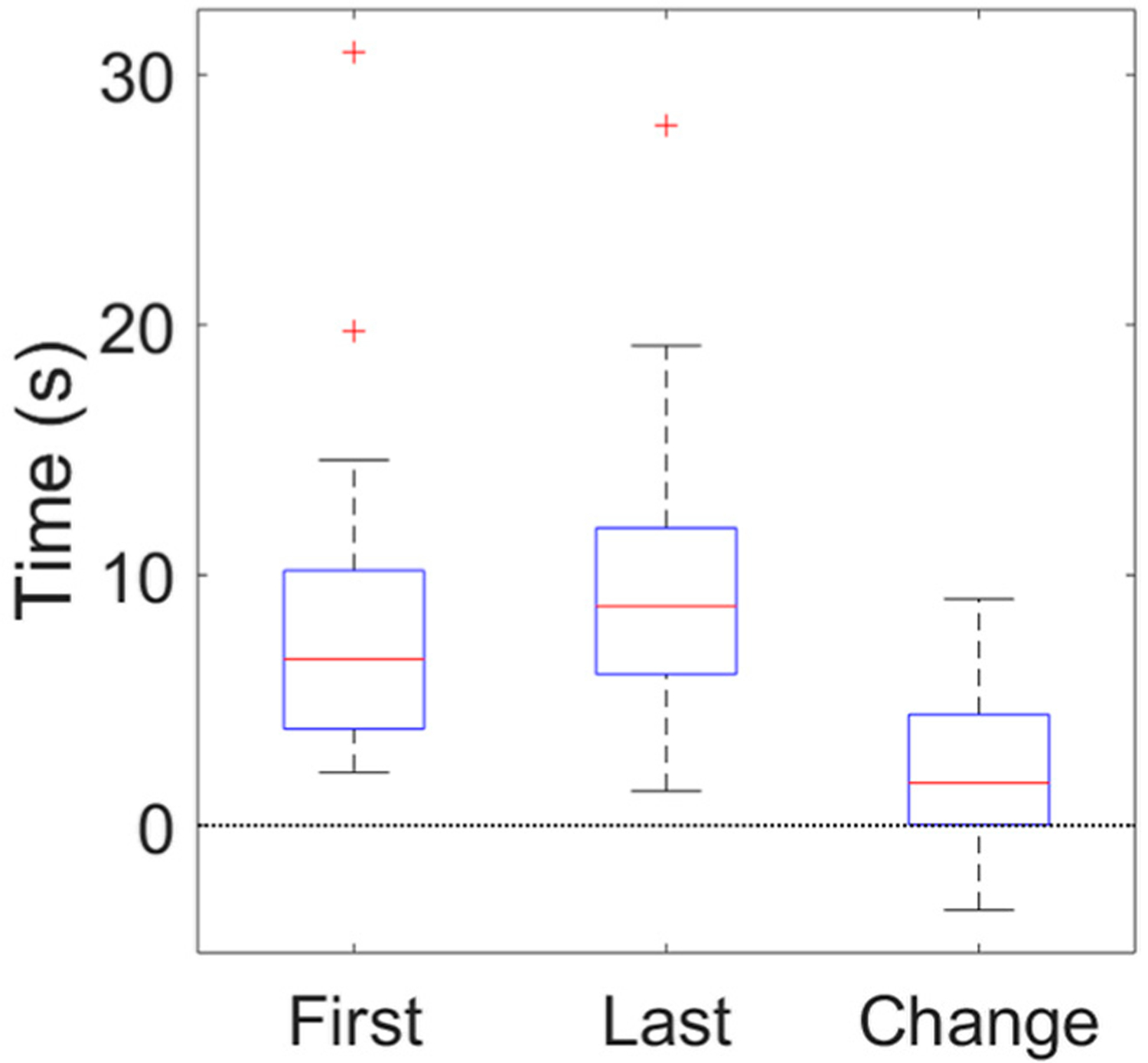
Distributions of microphone-based maximum phonation time for speakers’ first day before treatment (First), last day after treatment at discharge (Last), and first-to-last day changes (Change). Each box–whisker plot indicates median (horizontal line within box), interquartile range between 25th and 75th percentiles (box bounds), minimum value within 1.5 times the interquartile range away from the bottom of the box (lower whisker bound), maximum value within 1.5 times the interquartile range away from the top of the box (upper whisker bound), and outliers that exist outside these minimum and maximum values (+ symbols).

Figure 4 shows box-and-whisker plots of the statistically significant difference between admission and discharge MPTs, as well as the admission-to-discharge change in MPT. Notably, one participant had very high MPT at both admission (31 s) and discharge (28 s). Those MPTs are even longer than the mean MPT for this participant’s age group in [47]. The speaker’s voice was severely dysphonic, with a CPP of 4.9 dB at admission and 7.4 dB at discharge. These CPP values are both substantially below the clinical threshold of 14.45 dB identified in [48] and are very likely to indicate a voice disorder. Perceptually, the speaker had substantial strain and low airflow, which likely allowed them to conserve breath and produce a long MPT. For this participant, and any other speakers with similar voices, long MPT likely does not indicate a low decompensation risk. This study attempted to exclude participants with a current or history of a voice disorder. However, many voice issues are undiagnosed, and elderly speakers may be particularly unlikely to seek diagnosis or treatment [49]. In future data collection, it may be desirable to actively screen participants for voice disorders using some acoustic or auditory-perceptual criteria. Data from speakers with non-HF-related dysphonia should be interpreted with caution.

#### 4.1.3. Cepstral Peak Prominence

The effect size for average CPP, a standard indicator of vocal quality, in sustained vowels was comparatively small, at approximately 0.2. Inspecting the distribution of CPP values revealed that the average CPP at admission was below the normative threshold identified in [48]. Notably, those norms were computed on younger, comparatively healthy speakers relative to the HF population here, and future work is needed to identify CPP norms for older adults. Regardless, speakers whose CPP was low at admission were much more likely to show increases in CPP with treatment. Of speakers with CPP below the normative value at admission, 76% (13 of 17) had increased CPP at discharge. In contrast, of speakers with admission CPP above the normative value, only 47% (14 of 30) had increased CPP at discharge. The difference between the CPP changes in the low-CPP and high-CPP groups was nearly significant (paired *t*-test; *p* = 0.055). Future work could investigate other characteristics common to this low-CPP group and potentially identify speakers whose voices are likely to respond to changes in HF status.

Interestingly, CPP tended to decrease for continuous speech tasks. This finding is unexpected, especially in light of the increases in CPP for sustained vowels. Increases in the usage of creaky voice, which tended to occur for continuous speech but not sustained vowels, may contribute to the decline in CPP at discharge relative to admission.

### 4.2. Binary Classification Using Logistic Regression

The MPT− model’s predictions tended to be biased toward admission, with 54 admission predictions and 37 discharge predictions. The input data set was slightly biased towards admission recordings, with 47 recordings from admission and 44 from discharge. To adjust this bias in future work, it may be fruitful to adjust the probability threshold needed to predict discharge. Currently, any data point with discharge probability over 0.5 received a discharge prediction, but that 0.5 threshold could be optimized with more data. The MPT+ model’s predictions were more balanced than those of the MPT− model, with 22 admission and 20 discharge predictions. The MPT+ model’s predictions were also more accurate overall. This comparison suggests that MPT is a useful feature to identify decompensated heart failure and is worth investigating more closely in future work.

The feature weights and odds ratios provide information about each feature’s predictive power. Feature weights farther from 0 indicate that the model’s prediction is based relatively more on that feature. It is noted that results may be sensitive to the study’s regularization method applied to the model coefficients. The odds ratios for the MPT− model indicate that a discharge prediction was associated with increased mean F0 in vowels; greater phonatory stability (decreased F0 SD, increased CPP, and decreased CPP SD) in vowels; increased creak percent in sentences; faster speech rate in the Rainbow Passage; and reduced, more regular pauses in the Rainbow Passage and spontaneous speech. More specifically, in the Rainbow Passage, the percent of time devoted to speaking rather than pausing increased and the standard deviation of phrase durations decreased. The mean and median phrase duration both increased for spontaneous speech. These results broadly support our hypotheses that voice and speech characteristics at discharge would have reduced instability, increased F0, longer phrase durations, and less pausing.

In this study, as in [28], we found that post-treatment voices contained a higher proportion of creaky voice quality than did pre-treatment voices. This finding may be counterintuitive at first: creaky phonation is a form of vocal instability, and post-treatment voices tended to be more stable. However, creaky voice production requires the vocal fold superior layer to be slack, and vocal fold edema due to the presence of ADHF could stiffen the vocal folds and make that slackening more difficult to achieve. After treatment, when the edema is reduced, it is possible that creaky voice production may have returned to its higher, baseline characteristics.

The MPT+ model’s optimized λ was higher, so fewer features had non-zero weights after regularization. In this model, the probability of a recording being classified as at discharge was associated with increased F0 SD in sentences, increased MPT, and longer phrase durations in spontaneous speech. Notably, the MPT− model showed a *decrease* in F0 SD for sustained vowels, while the MPT+ model showed an increase in F0 SD in CAPE-V sentences. These divergent changes might appear contradictory but are likely driven by different mechanisms. In sustained vowel phonation, the speaker generally maintains a stable F0 target, although even healthy speakers naturally produce some variation around the target. For sustained vowels, F0 SD reflects that variation around the F0 target, and its decrease with treatment indicates that healthier speakers had more stable phonation. For continuous speech, there is no single F0 target; instead, healthy speakers produce a wide F0 range as their pitch varies due to prosody. Speakers at discharge exhibited increased F0 SD during continuous speech, indicating a more flexible, wider use of their prosodic range.

### 4.3. Day-to-Day Trajectories of Model Performance

The day-to-day trajectories of the discharge probability were shown in Figure 3. They reveal that, in general, most participants showed an increased probability of discharge on their last day compared to their first. This increase occurred for most participants even though it did not always cross the category boundary from an admission prediction to a discharge prediction. The MPT− model showed more day-to-day variability than the MPT+ model did, possibly because it incorporated more features. In the MPT− model, 49 participants had usable data from more than one recording session. Of those 49 participants, 39 (80%) had an increased discharge probability on their last day compared to their first day. This result suggests that future work could investigate appropriate thresholds for distinguishing voices with and without decompensation. In theory, the classification could be related to the change in discharge or admission probability rather than an absolute threshold. In other words, for a patient being monitored at home, a certain level of increase in the probability of admission could trigger an alert to care providers.

Notably, many day-to-day trajectories were not monotonic, i.e., they had repeated fluctuations in the discharge probability. Alternately, for some speakers, the trajectory involved several stable days followed by a dramatic change in discharge probability and then more stable days. The trends may be linear, monotonic but nonlinear, or fluctuating, depending on the particular situation of a patient. In future work, these fluctuations could be correlated with weight changes or medication dosages to determine a more exact relationship between fluid level and voice and speech biomarkers.

### 4.4. Implications of Accelerometer-Based Results

For some acoustic measures (e.g., maximum phonation time, F0-related measures, and CPP-related measures in continuous speech), effect sizes tended to be similar for the ACC-based data and MIC-based data (Table 1). In contrast, several of the large effect sizes for speech phrase-related measures in the MIC data were not comparably large for the ACC data. For example, total phrase duration had an effect size of −0.5 for both the MIC-based Rainbow Passage and second reading passage, whereas for the ACC-based Rainbow Passage and second reading passage, the effect sizes were lower at −0.33 and −0.22, respectively. The speech phrase measures were computed by identifying voiced and unvoiced regions in the speech signals, so differences in the behavior of the pitch-tracking algorithms for MIC and ACC signals could have contributed to this difference.

Logistic classifier performance was broadly similar for the MIC-based and ACC-based models (Figure 2). The MPT+ model performed very similarly with both MIC and ACC data, while the MPT− model had somewhat lower test accuracy with ACC data compared to MIC. Both models preserved similar features for MIC and ACC input. The most predictive features in the ACC-based MPT− model related to an increased F0 mean; greater phonatory stability (reduced CPP SD, and increased HNR); increased creaky voice percent; faster speech; and less-frequent pauses. Like the MIC-based model, the ACC-based MPT+ model showed that increased MPT and F0 SD in CAPE-V sentences were related to an increased discharge probability.

### 4.5. Future Work

Suggestions for future work that are raised by specific results were discussed above. More broadly, future work could look at subgroups of the already enrolled participants to identify whether there are non-voice factors that affect how voice responds to HF status. For example, any effects of age, sex, HF subtype (systolic or diastolic failure), or previous medical history could all be relevant. Additionally, data from the intermediate recordings (between admission and discharge) could be used to train a linear model that predicts, for example, how many days from admission a sample was recorded on. In contrast to the logistic models presented here, predicting a continuous dependent variable could provide more information about the trajectory of voice changes during treatment for HF.

This study examined voice and speech changes in patients recovering from decompensated HF. In future data collection, it would be useful to collect data from HF patients who are not decompensated but are at risk of developing ADHF. For example, the 30-day readmission rate after an episode of ADHF has been reported to be approximately 24% [8]. Analyzing voice and speech patterns from patients who have just recovered from ADHF could be used to predict readmission. This prospective monitoring could also test hypotheses in the preventative direction (starting with stable patients and attempting to predict decompensation) rather than following patients as their HF status improved in this study. Monitoring discharged patients may also provide a clearer idea of how closely voice changes leading up to decompensation actually mirror voice changes during treatment.

## 5. Conclusions

Acoustic voice and speech features show promise as biomarkers for ADHF, possibly because systemic fluid accumulation in the lungs and larynx affect phonation and respiration. In this study, potential vocal biomarkers for ADHF were identified and evaluated based on a set of spoken recordings from patients receiving inpatient ADHF treatment. Logistic regression models were trained to classify recordings as pre-treatment (at admission) or post-treatment (at discharge). For each recording, these models output a probability that the recording was produced at discharge rather than at admission, with a classification accuracy as high as 69%. Additionally, the probability of discharge increased from the pre-treatment voice sample to the post-treatment sample for up to 80% of participants. Voice and speech characteristics at discharge were associated with longer maximum phonation times, more stable phonation, increased creak percent, faster speech rate, and reduced pausing compared to acoustic characteristics at admission for the same individuals.

## Figures and Tables

**Table 1.** Effect size (paired Cohen’s d) for selected features computed from the acoustic microphone (MIC) and neck-surface accelerometer (ACC) from the given speech tasks, sorted in decreasing order of MIC-based effect size magnitude.

Feature	Task	Effect Size (MIC)	Effect Size (ACC)
Total phrase duration	Rainbow Passage	−0.50	−0.33
Total phrase duration	2nd passage	−0.50	−0.22
Phonation time	Max phonation	0.49	0.45
F0 mean	Vowel	0.35	0.35
CPP SD	Sentences	−0.27	−0.30
CPP mean	Sentences	−0.25	−0.31
CPP median	Sentences	−0.22	−0.28
CPP median	Vowel	0.21	−0.018
CPP SID	Vowel	−0.17	−0.29
CPP mean	Vowel	0.17	−0.038

**Table 2.** Odds ratios for the MPT− models derived separately for the microphone (MIC) and neck-surface accelerometer (ACC) sensors.

Feature	Task	Odds Ratio (MIC)	Odds Ratio (ACC)
Creak %: 0.3 threshold	Sentences	1.92	
F0 mean	Vowel	1.40	1.08
CPP median	Spontaneous	1.37	
Phrase duration median	Spontaneous	1.27	
Phrase duration mean	Spontaneous	1.25	1.13
Phrase %	Rainbow Passage	1.18	
Creak %: 0.02 threshold	Sentences		1.12
CPP median	Vowel	1.08	
Phrase %	Spontaneous		1.08
Harmonics-to-noise ratio	Vowel		1.07
CPP SD	Rainbow Passage	0.92	
Phrase count	Rainbow Passage		0.91
CPP mean	Sentences		0.90
Phrase duration SD	Rainbow Passage	0.87	
F0 SD	Vowel	0.85	
F0 SD	Spontaneous	0.79	
Total phrase duration	Rainbow Passage	0.70	0.92
CPP SD	Sentences	0.61	0.85

**Table 3.** Feature weights and odds ratios for the MPT+ model.

Feature	Task	Odds Ratio (MIC)	Odds Ratio (ACC)
Phonation time	Max phonation	1.34	1.03
F0 SD	Sentences	1.25	1.02
Phrase duration median	Spontaneous	1.11	

**Table 4.** Classification performance metrics reported for each of five logistic classifier models using microphone (accelerometer) recordings.

λ	Model	Accuracy	AUC	TAR	TDR	APV	DPV
N/A	MPT−	0.53 (0.53)	0.49 (0.53)	0.51 (0.49)	0.55 (0.57)	0.55 (0.54)	0.51 (0.52)
N/A	MPT+	0.52 (0.58)	0.46 (0.54)	0.48 (0.50)	0.57 (0.65)	0.53 (0.59)	0.52 (0.57)
N/A	MPT-only	0.64 (0.58)	0.61 (0.60)	0.63 (0.52)	0.65 (0.64)	0.63 (0.57)	0.65 (0.59)
L1	MPT−	0.62 (0.56)	0.66 (0.51)	0.70 (0.60)	0.52 (0.52)	0.61 (0.56)	0.62 (0.56)
L1	MPT+	0.69 (0.68)	0.65 (0.63)	0.71 (0.70)	0.67 (0.65)	0.68 (0.67)	0.70 (0.68)

AUC = area under the receiver operating characteristic curve, TAR = true admission rate (sensitivity), TDR = true discharge rate (specificity), APV = admission predictive value (positive predictive value), DPV = discharge predictive value (negative predictive value), N/A = not applicable for models without regularization, L1 = L1 regularization applied, MPT = maximum phonation time, MPT− = model without MPT, MPT+ = model with MPT, MPT-only = model with MPT only.

## Data Availability

Mass General Brigham and Mass General are not allowed to give access to data without the Principal Investigator (PI) for the human studies protocol first submitting a protocol amendment to request permission to share the data with a specific collaborator on a case-by-case basis. This policy is based on very strict rules dealing with the protection of patient data and information. Anyone wishing to request access to the data must contact the corresponding author.
